# The Physical Genome Mapping of *Anopheles albimanus* Corrected Scaffold Misassemblies and Identified Interarm Rearrangements in Genus *Anopheles*

**DOI:** 10.1534/g3.116.034959

**Published:** 2016-11-07

**Authors:** Gleb N. Artemov, Ashley N. Peery, Xiaofang Jiang, Zhijian Tu, Vladimir N. Stegniy, Maria V. Sharakhova, Igor V. Sharakhov

**Affiliations:** * Laboratory for Ecology, Genetics, and Environmental Protection, Tomsk State University, 634050, Russia; † Department of Entomology and Fralin Life Science Institute, Virginia Polytechnic Institute and State University, Blacksburg, Virginia 24061; ‡ The Interdisciplinary PhD Program in Genetics, Bioinformatics, and Computational Biology, Virginia Polytechnic Institute and State University, Blacksburg, Virginia 24061; § Department of Biochemistry and Fralin Life Science Institute, Virginia Polytechnic Institute and State University, Blacksburg, Virginia 24061

**Keywords:** mosquito genome, fluorescence *in situ* hybridization, physical mapping, polytene chromosomes, *Anopheles albimanus*

## Abstract

The genome of the Neotropical malaria vector *Anopheles albimanus* was sequenced as part of the 16 *Anopheles* Genomes Project published in 2015. The draft assembly of this species consisted of 204 scaffolds with an N50 scaffold size of 18.1 Mb and a total assembly size of 170.5 Mb. It was among the smallest genomes with the longest scaffolds in the 16 *Anopheles* species cluster, making *An. albimanus* the logical choice for anchoring the genome assembly to chromosomes. In this study, we developed a high-resolution cytogenetic photomap with completely straightened polytene chromosomes from the salivary glands of the mosquito larvae. Based on this photomap, we constructed a chromosome-based genome assembly using fluorescent *in situ* hybridization of PCR-amplified DNA probes. Our physical mapping, assisted by an ortholog-based bioinformatics approach, identified and corrected nine misassemblies in five large genomic scaffolds. Misassemblies mostly occurred in junctions between contigs. Our comparative analysis of scaffolds with the *An. gambiae* genome detected multiple genetic exchanges between pericentromeric regions of chromosomal arms caused by partial-arm translocations. The final map consists of 40 ordered genomic scaffolds and corrected fragments of misassembled scaffolds. The *An. albimanus* physical map comprises 98.2% of the total genome assembly and represents the most complete genome map among mosquito species. This study demonstrates that physical mapping is a powerful tool for correcting errors in draft genome assemblies and for creating chromosome-anchored reference genomes.

*Anopheles albimanus* is one of the main malaria vectors in the Americas (Sinka *et al.* 2010). This species is distributed in the Neotropical region stretching from the southern United States to northern Peru and the Caribbean Islands. It is the major contributor to malaria transmission in the coastal areas of this region. Like most other species from this Neotropical region, *An. albimanus* is a member of subgenus Nyssorhynchus, a lineage resulting from one of the earliest radiations within the *Anopheles* genus (Harbach and Kitching 2016). Because of its importance in malaria transmission and the availability of a robust laboratory colony, genetics and cytogenetics of *An. albimanus* have been studied for decades. Like other mosquitoes from the genus *Anopheles*, *An. albimanus* has highly polytenized chromosomes in salivary glands of larvae (Hobbs 1962). The first drawn cytogenetic map with a detailed description of chromosomal banding patterns for this species was developed in 1973 (Keppler *et al.* 1973). The first successful *in situ* hybridization of histone genes with chromosomes of *An. albimanus* was performed in 1993 (Narang and Seawright 1993). A cytogenetic photomap for the salivary gland polytene chromosomes of *An. albimanus* was used for chromosomal localization of 17 DNA probes from *An. gambiae* (Cornel and Collins 2000). Cytogenetic studies facilitated the creation of a genetic sexing strain with a radiation-induced Y chromosome–2R translocation and 2R inversion (Kaiser *et al.* 1978). The first genetic linkage map for *An. albimanus* was constructed in 1989 using morphological mutants and biochemical markers (Narang and Seawright 1989). A new genetic map was recently generated using microsatellite markers (Penilla *et al.* 2009).

*An. albimanus* has several unique traits that distinguish this species from other malaria mosquitoes and make it highly suitable for genome sequencing projects. Unlike other species from genus *Anopheles*, which usually belong to species complexes, no evidence for cryptic species of this vector has been described (Narang *et al.* 1991; Arredondo-Jimenez *et al.* 1992). Also, chromosomes of *An. albimanus* typically lack polymorphic inversions, which other *Anopheles* species are renowned for (Coluzzi *et al.* 2002; Pombi *et al.* 2008; Ayala *et al.* 2014). For example, a comprehensive cytogenetic study of samples from 11 distant localities in Colombia found only one small low-frequency inversion on chromosome X in certain populations (Narang *et al.* 1991). The lack of sibling species and reduced chromosomal polymorphism may contribute to a low overall heterozygosity of the *An. albimanus* samples from which the reads were obtained to build the genome assembly. In addition, *An. albimanus* has one of the smallest genome sizes (170.5 Mb) and the lowest repeat content (only 2%) among mosquito species (Neafsey *et al.* 2015; Sharakhov and Sharakhova 2015). The *An. albimanus* genome was sequenced as part of the 16 *Anopheles* Genomes Project that generated a resource for hypothesis testing to increase our understanding of genetic determinants of vectorial capacity (Neafsey *et al.* 2013, 2015). The *An. albimanus* assembly was made from 101-bp paired-end Illumina HiSeq2000 reads generated from three libraries: a 180-bp insert “fragment” library, a 1.5-kb “jump” library, and a 38-kb fosmid scale illumina (“fosill”) library. Reads were assembled using the ALLPATHS LG algorithm, with the Haploidify option to reduce haplotype assemblies caused by high heterozygosity. Reduced heterozygosity and low-repeat content were among the main factors that determined the greatest length of genomic scaffolds in *An. albimanus* compared with other species. The *An. albimanus* genome assembly resulted in 170,508,315 bp consisting of 204 scaffolds with an N50 scaffold size of 18,068,499 bp, the longest N50 value among all sequenced mosquito genomes in this project (Neafsey *et al.* 2015).

The development of chromosome-based assemblies for eukaryotic genomes corrects scaffold arrangements and makes possible studies of chromosome organization and evolution. For example, two independent physical mapping methods, fluorescence *in situ* hybridization (FISH) and optical mapping, have determined the linear genome organization and corrected the arrangement of 45 scaffolds mostly in pericentric heterochromatin of tomato *Solanum lycopersicum* (Shearer *et al.* 2014). A recent FISH mapping of genomic scaffolds to polytene chromosomes of *Drosophila willistoni* has reassigned chromosome arms IIL and IIR to Muller elements B and C (Garcia *et al.* 2015). A combination of linkage mapping with Pacific Biosciences long-read sequencing has allowed anchoring 84% of the *Heliconius melpomene* genome onto chromosomes and confirmed 10 chromosome fusions in 6 million years of butterfly evolution (Davey *et al.* 2016). Long genomic scaffolds facilitate anchoring the *An. albimanus* assembly to chromosomes via physical mapping. Our recent study utilized previously mapped markers (Cornel and Collins 2000) and newly mapped DNA probes to develop a physical map covering 75% of the *An. albimanus* genome (Neafsey *et al.* 2015). A gene order comparison has been conducted between *An. gambiae* and species with partially mapped genome assemblies including *An. albimanus*, *An. atroparvus*, *An. funestus*, and *An. stephensi*. The analysis supported the previous findings that chromosomal arms in *Anopheles* reshuffle between chromosomes via whole-arm translocations (Green and Hunt 1980; Cornel and Collins 2000; Sharakhov *et al.* 2001, 2002; Xia *et al.* 2010; Sharakhova *et al.* 2011, 2013, 2014; Jiang *et al.* 2014; Liang *et al.* 2014; Artemov *et al.* 2015). It has also shown that, unlike *Drosophila*, mosquito chromosomes do not undergo fission or fusion. The study found numerous paracentric inversions within chromosomal arms but no pericentric inversions or partial-arm translocations. Finally, the work revealed that the sex chromosome, X, has the highest rate of inversion fixation among chromosomal arms and the highest rate of gene movement to other chromosomes (Neafsey *et al.* 2015).

Here we report a new detailed cytogenetic photomap and a chromosomally anchored genome assembly covering 98.2% of the *An. albimanus* genome, which is the most complete chromosomal genome assembly for any mosquito to date (*e.g.*, compared to 84.3% of *An. gambiae*). Our work demonstrates that physical mapping can be effectively used for correcting misassemblies in sequenced genomes. The new genome map can be used as a reference for population genomics studies of this Neotropical malaria vector and for exploration of chromosomal evolution in malaria mosquitoes.

## Materials and Methods

### Mosquito strain and larvae preservation

The STECLA strain of *An. albimanus* was maintained in the insectary of the Fralin Life Science Institute, Virginia Tech. The strain was originally colonized from an El Salvador population and deposited at the Malaria Research and Reference Reagent Resource (MR4) at the Biodefense and Emerging Infections Research Resources Repository (*BEI*) under catalog number MRA-126. Larvae were raised in a growth chamber at 27°, with a 12-hr cycle of light and darkness_._ Fourth instar larvae were fixed in cold Carnoy’s solution (3 ethanol: 1 glacial acetic acid by volume) at −20° for at least 2 wk prior to dissection.

### Chromosome preparation and cytogenetic map development

Salivary glands dissected from one or two 4th instar larvae were used for one chromosome preparation. Isolated salivary glands were bathed in a drop of 50% propionic acid for 5 min and squashed as previously described (Sharakhova *et al.* 2015). The quality of the preparation was assessed with an Olympus CX41 phase-contrast microscope (Olympus America Inc., Melville, NY). High-quality chromosome preparations were then flash frozen in liquid nitrogen and immediately placed in cold 50% ethanol. After that, preparations were dehydrated in an ethanol series (50, 70, 90, and 100%) and air-dried. Unstained chromosomes were observed using an Olympus BX41 phase-contrast microscope with attached CCD camera Qcolor5 (Olympus America Inc., Melville, NY). For the chromosome map development, about 200 images of well-polytenized and well-spread chromosomes were obtained. Images were combined, straightened, shaped, and cropped using AdobePhotoshop CS2 software. The chromosome nomenclature was adopted from the previously published cytogenetic maps of *An. albimanus* (Keppler *et al.* 1973; Cornel and Collins 2000).

### Probe preparation and FISH

Gene-specific primers were designed to amplify unique exon sequences from the beginning and end of each scaffold using the primer-BLAST program (Ye *et al.* 2012) available at the National Center for Biotechnology Information (NCBI) (http://www.ncbi.nlm.nih.gov/tools/primer-blast/). The primer design was based on gene annotations from the AalbS1 genome assembly available at VectorBase (https://www.vectorbase.org/organisms/anopheles-albimanus/stecla/aalbs1) (Giraldo-Calderon *et al.* 2015). PCR was performed using 2X Immomix DNA polymerase (Bioline USA Inc., MA) and a standard Immomix amplification protocol. Amplified fragments were labeled with Cy3 and Cy5 fluorescent dyes (GE Health Care, UK Ltd, Buckinghamshire, UK and Enzo Biochem, Enzo Life Sciences Inc., Farmingdale, NY) or TAMRA-5-dUTP (Biosan, Novosibirsk, Russia) using a Random Primers DNA Labeling System (Invitrogen, Carlsbad, CA). FISH was performed according to the previously described standard protocol (Sharakhov 2015). DNA probes were hybridized to the chromosomes at 39° during 10–15 hr in a hybridization solution (50% formamide; 10% sodium dextransulfate, 0.1% Tween 20 in 2XSSC, pH 7.4). Chromosome preparations were washed in 0.2XSSC (saline-sodium citrate: 0.03 M sodium chloride, 0.003 M sodium citrate) and counterstained with DAPI in ProLong Gold Antifade Mountant (Thermo Fisher Scientific Inc.).

### Bioinformatics approaches

A Hidden Markov model (HMM) was used to predict misassemblies in the *An. albimanus* genome assembly. The HMM was built based on the probability of each ortholog presented on each chromosomal element. Genome assemblies and annotations of 16 mosquito species were downloaded from VectorBase (Giraldo-Calderon *et al.* 2015) and *Anophelinae* orthology information was obtained from OrthoDB (Waterhouse *et al.* 2013). The emission probability matrix for HMM was constructed as follows: the probability of location of each ortholog on each chromosomal element was estimated based on its chromosomal location in *An. gambiae*; if the estimated value was zero, the value was set to an arbitrary value (0.05). The chance of misassembly occurring between two neighboring genes was represented by a state transition rate. The state transition rate was set to be 0.0001 to build a transition probability matrix. The Viterbi algorithm was used to predict the most likely chromosomal element each gene is on. Scaffolds with genes assigned to more than one chromosomal element were considered potentially misassembled. The prediction was performed with the R Package HMM environment (Himmelmann 2010).

A BLAST tool (www.vectorbase.org/blast) available at VectorBase (Giraldo-Calderon *et al.* 2015) was used to find orthologs of *An. albimanus* genes in the *An. gambiae* and *An. atroparvus* genomes. These genes were utilized for adjusting the boundaries of misassembled scaffolds. The BLAST tool was also applied to localize microsatellites in the *An. albimanus* genome and to compare the physical and genetic maps of *An. albimanus* (Penilla *et al.* 2009).

### Data availability

All data are available in the main paper and in Supplemental Material, Table S1, Table S2, Table S3, Table S4, and Table S5. VectorBase has handled the public release of the *An. albimanus* chromosomal genome assembly, and the data will be made available via the VectorBase December 2016 release (https://www.vectorbase.org) (Giraldo-Calderon *et al.* 2015).

## Results

### A high-resolution cytogenetic photomap for An. albimanus

**Figure 1:**
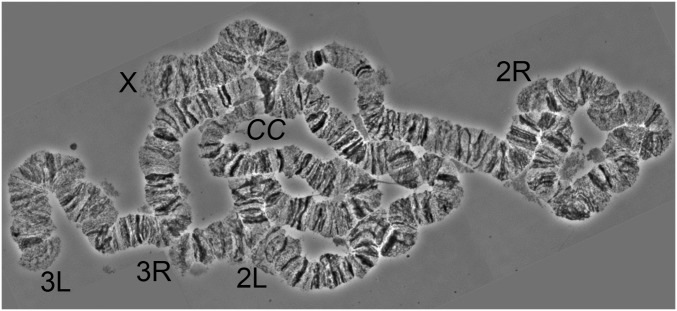
A phase-contrast image of well-polytenized chromosomes from salivary glands of *An. albimanus* larva. Chromosome arm names are indicated as X, 2R, 2L, 3R, and 3L; the chromocenter is shown as *CC*.

**Figure 2:**
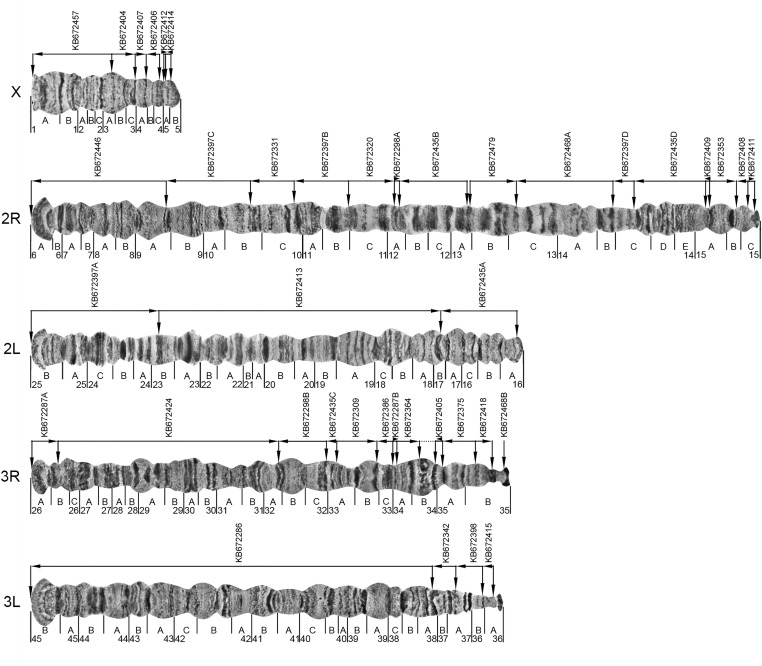
A high-resolution cytogenetic map and physical genome map for *An. albimanus*. Numbered divisions and lettered subdivisions are shown below the chromosome images. Horizontal lines and arrows indicate the order and orientation of genomic scaffolds. The names of genomic scaffolds are shown above horizontal lines. Fragments of misassembled scaffolds are marked by a scaffold name followed by a capital letter. The start and end positions of the genomic scaffolds are shown by vertical arrows corresponding to mapped FISH probes. The dotted horizontal line in 3R:34B indicates a predicted adjacency of scaffolds KB672364 and KB672405.

*An. albimanus* exhibits a chromosomal complement typical for *Anopheles* where 2*n* = 6. The chromosomes are represented by two pairs of metacentric autosomes and one pair of subtelocentric sex chromosomes (Hobbs 1962). Because of homologous pairing, polytene chromosomes in salivary glands of *An. albimanus* are represented by five chromosomal arms: the smallest X chromosome, the longest 2R arm, and the almost equal in length 2L, 3R, and 3L arms. The heterochromatic Y chromosome does not polytenize. The pericentromeric regions of polytene chromosomes are usually bound together in the chromocenter (Figure 1), but sometimes the short X chromosome dissociates from the autosomes. In this study, we constructed a cytogenetic photomap using phase-contrast images of unstained well-polytenized chromosomes from salivary glands of *An. albimanus*. Fine details of the chromosomal structure, including patterns of thin and light bands, are clearly visible in this high-resolution map. Chromosomes are completely straightened to facilitate physical genome mapping (Figure 2). To further assist in the recognition of a banding pattern, we provide a detailed description of major cytogenetic landmarks for all chromosomal arms. Chromosomes are divided into 45 numbered divisions and 110 lettered subdivisions. The division borders and nomenclature are adopted from the previously published drawn map (Keppler *et al.* 1973) and photomap (Cornel and Collins 2000) of *An. albimanus*.

Chromosomal arms of *An. albimanus* have regions with a reproducible distinct morphology called “landmarks” that can be used for arm recognition. The lengths of the X chromosome and 2R arm make them easily identifiable as the shortest and longest arm, respectively. Additional landmarks for the X chromosome are a bell-shaped telomere end with a pair of dark bands in the middle of region 1A and a puffy area in region 3A. All autosomal telomeres of *An. albimanus* have flared ends with only slight differences in morphology, thus they cannot serve as good landmarks for arm identification. Pericentromeric regions are usually underpolytenized and not properly spread due to the formation of the chromocenter. For these reasons, we rely on internal chromosomal regions for arm identification in *An. albimanus*. Three thin bands in region 7A, as well as a dark thick band surrounded by thin bands in region 15B, are robust landmarks for arm 2R. Despite their nearly equal length, the 2L, 3R, and 3L arms can be easily recognized by distinct landmarks in the middle of the arms. A pair of thick double-bands in 17AB and a wide dark band in region 20B are landmarks for arm 2L. Both arms of chromosome 3 have relatively thin, cone-shaped pericentromeric regions in 35B and 36AB. The major landmark for arm 3R is a wide granulated band surrounded by two dark bands in region 34B. A series of three dark bands surrounded by light areas in region 30A and a wide dark band in region 28B can be used as additional landmarks for 3R. In some specimens, we observed a large puff in region 31AB. If present, this puff can also be utilized as a strong landmark for 3R. Arm 3L can be recognized by a pair of very distinct dark bands in region 37A. Additional landmarks for 3L are a light puffy area in region 38C–39A surrounded by strong bands in regions 38B and 39B (Figure 2).

### Identification and correction of misassemblies in genomic scaffolds

**Figure 3:**
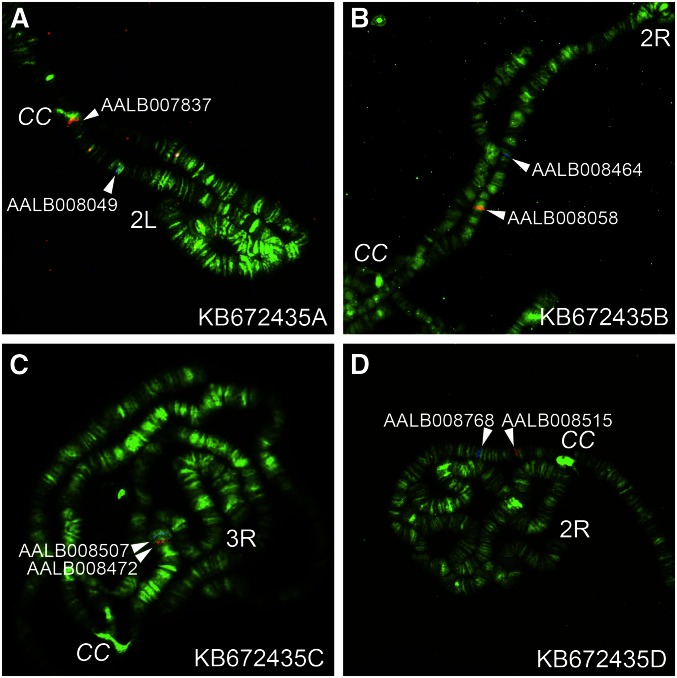
Physical mapping of misassembled fragments in scaffold KB672435. Fragments KB672435A, KB672435B, KB672435C, and KB672435D are found in 2L (A), in 2R (B), in 3R (C), and in 2R (D), respectively.

Our physical mapping, assisted by the HMM approach and synteny analysis, identified and corrected a total of nine misassemblies within five large scaffolds (Table 1 and Table S1). The lengths of original misassembled scaffolds were 2.9, 3.1, 6.2, 15.4, and 24.1 Mb, with a total length of 51.6 Mb. We identified and corrected the misassemblies in four steps. First, we detected misassembled scaffolds by physically mapping the outmost genes to unexpectedly different locations on chromosomes. Second, we used an HMM approach to narrow down the misassembly boundaries within mapped scaffolds and to predict new misassemblies. Third, we performed FISH with chromosomes to validate misassemblies predicted by the HMM method. Finally, we manually investigated and adjusted misassembly boundaries by analyzing adjacencies of orthologous genes in the *An. gambiae* and *An. atroparvus* genomes. We used *An. gambiae* for our synteny analysis because it has a chromosome-based genome assembly and belongs to the subgenus *Cellia*. We also used *An. atroparvus* because it has relatively long scaffolds and belongs to the subgenus *Anopheles*. Our study found that eight out of nine cases of misassembly occurred in gaps between genomic contigs that were erroneously bridged within scaffolds. One misassembly happened within contig APCK01000384.1 of scaffold KB672397 in the area that contains a cluster of 12 transfer RNA (tRNA) genes. The cluster consists of genes encoding six tRNAs-Ile, two tRNAs-Gly, two tRNAs-Trp, one tRNA-Tyr, and one tRNAs-Lys. It is worth noting that KB672397 is the second longest scaffold in the AalbS1 assembly. In fact, the five scaffolds identified with misassemblies are all in the top 11 longest scaffolds. We named each misassembled fragment within a scaffold as a new scaffold by adding a capital letter to the end of the existing name. For example, scaffold KB672435 (15.4 Mb) was composed of four misassembled fragments that localized to two places on 2R, one location on 2L, and one on 3R (Figure 3). We named these fragments KB672435A (3.4 Mb), KB672435B (7.2 Mb), KB672435C (0.9 Mb), and KB672435D (3.7 Mb). Most of the identified misassemblies were predicted by the HMM bioinformatics approach, which is based on *in silico* mapping of orthologs in genomes of other anophelines and in chromosomal elements of *An. gambiae*. We use the term “chromosomal elements” to define chromosomal arms that are homologous across species (Green and Hunt 1980; Sharakhova *et al.* 2013). Accordingly, the chromosomal arms in *An. gambiae* are named as follows: X = element 1 (e1), 2R = e2, 2L = e3, 3R = e4, and 3L = e5. In *An. albimanus* the correspondence between arms and chromosomal elements is as follows: X = e1, 2R = e2, 2L = e4, 3R = e5, and 3L = e3. The HMM prediction identified only misassemblies that fused genomic sequences from different chromosomal elements into one scaffold. The HMM method could not identify misassembled scaffolds with erroneously merged genomic sequences from the same chromosomal element, such as KB672287A and KB672287B, which both mapped to e5 (Table 1 and Table S1).

**Table 1:** Misassemblies within the *An. albimanus* genome

Fragments of Misassembled Scaffold	Size (bp)	Coordinates Within Original Scaffolds	Chromosomal Location	Orientation	Predicted by HMM
KB672287A	2,754,385	1–2,754,385	3R:26AB	+	No
KB672287B	360,489	2,754,680–3,115,168	3R:34A	+	No
KB672298A	426,774	1–426,774	2R:12A	+	Yes
KB672298B	2,468,322	427,598–2,895,919	3R:32AC	−	Yes
KB672397A	11,932,447	1–11,932,447	2L:23B-25B	−	Yes
KB672397B	4,937,263	11,985,224–16,922,486	2R:10C-11C	−	No
KB672397C	6,174,178	16,942,420–23,116,597	2R:9A-10B	−	No
KB672397D	910,984	23,155,150–24,066,133	2R:14BC	−	No
KB672435A	3,408,218	1–3,408,218	2L:16A-17B	+	Yes
KB672435B	7,248,733	3,505,816–10,754,548	2R:12A-13A	−	Yes
KB672435С	883,903	10,761,531–11,645,433	3R:33A	−	Yes
KB672435D	3,727,725	11,655,887–15,383,611	2R:14C-15A	−	Yes
KB672468A	5,915,397	1–5,915,397	2R:13C-14B	−	Yes
KB672468B	136,759	6,032,887–6,169,645	3R:35B	?	Yes

### Discovery of interarm rearrangements in genus Anopheles

**Figure 4:**
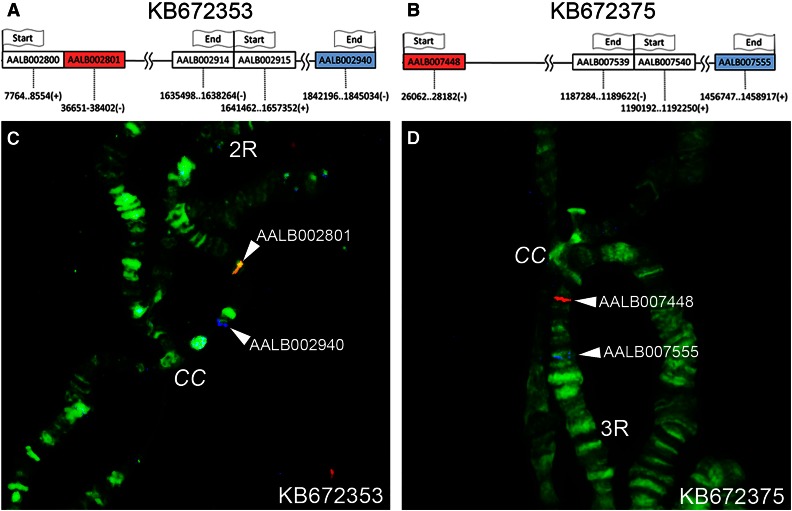
Results of the HMM analysis and FISH mapping of scaffolds KB672353 and KB672375. Flags mark the start and the end positions of bioinformatically predicted “misassembled” fragments of genomic scaffolds KB672353 (A) and KB672375 (B). Colored boxes in (A and B) indicate positions of the genes used in FISH. FISH results demonstrate the lack of misassemblies in scaffolds KB672353 (C) and KB672375 (D).

**Figure 5:**
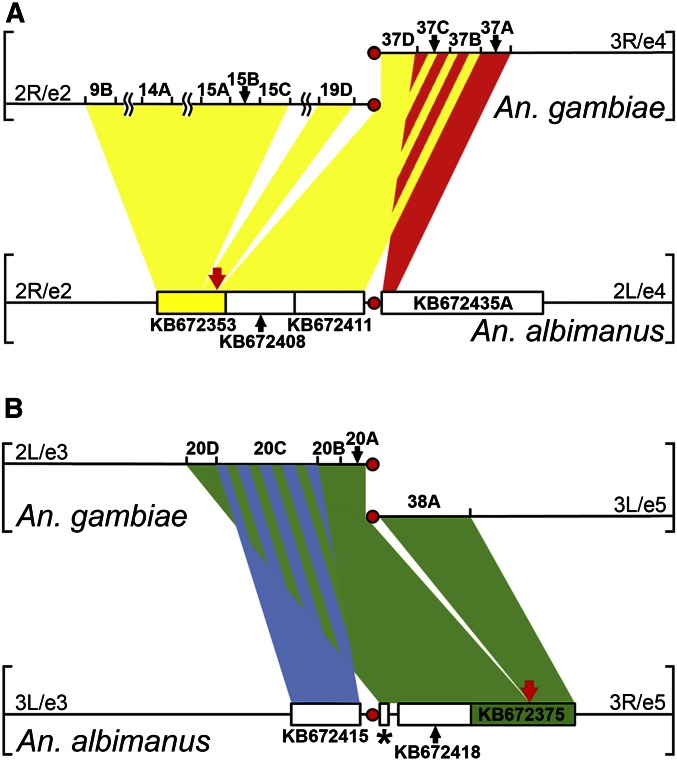
Localization of orthologous genes in pericentromeric regions of *An. gambiae* and *An. albimanus*. (A) The yellow and red shades demonstrate positions of orthologous genes in the e2 and e4 pericentromeric regions. (B) The blue and the green shades indicate gene positions in the e3 and e5 pericentromeric regions. Red arrows show predicted positions of rearrangement breakpoints in scaffolds KB672353 (A) and KB672375 (B). Red circles represent centromeres. The asterisk indicates scaffold KB672468B. Black arrows show locations of scaffolds and chromosome regions.

A total of eight potential misassemblies in six scaffolds were identified by the HMM approach alone (Table S2). Interestingly, two of these predicted misassemblies in scaffolds KB672353 and KB672375 were not confirmed by physical mapping. The HMM approach showed that KB672353 is divided into two different fragments: one of them (from gene AALB002800 to gene AALB002914) corresponds to the 2R arm (e2), and another one (from AALB002915 to AALB002940) corresponds to the 2L arm (e4). Similarly, one part of KB672375 (from AALB007448 to AALB007539) is expected to be on 3L chromosome (e3), and another one (from AALB007540 to AALB007555) is expected to be on 3R chromosome (e5). The HMM method is based on *in silico* mapping of orthologs to genomic scaffolds of other *Anopheles* species and to chromosomal elements of *An. gambiae*. The method assumes that the genomic organization of chromosome elements is exactly the same across species. Therefore, each identified discrepancy in a chromosomal assignment of a scaffold’s fragments is considered a misassembly. However, our FISH mapping has shown that the “misassemblies” identified in scaffolds KB672353 and KB672375 by this bioinformatics approach are not real. We mapped KB672353 and KB672375 entirely within single chromosomal elements 2R (e2) and 3R (e5), respectively (Figure 4). We concluded that the discrepancies between chromosome mapping of orthologous genes in *An. albimanus* and *An. gambiae* are caused by real rearrangements between the species. We analyzed genomic and chromosomal positions of genes located near breakpoints of these rearrangements in *An. albimanus* and *An. gambiae*. Our BLAST search localized the majority of KB672353 to the pericentromeric region of 2R arm (e2) of *An. gambiae*. However, sequences homologous to the short fragment of KB672353, as well as neighboring scaffolds KB67408 and KB672411, were found in chromosome element 4 (3R) of *An. gambiae* where they intermingled with sequences homologous to KB672435A from element 4 (Figure 5A). Scaffold KB672375 was localized in 3R/e5 of *An. albimanus*, but only a small fragment of KB672375 was homologous to 3L/e5 of *An. gambiae*. The long fragment of KB672375, as well as neighboring scaffolds KB672418 and KB672468B from 3R/e5 of *An. albimanus*, were homologous to 2L/e3 of *An. gambiae* where their sequences intermingled with sequences homologous to KB672415 from element 3 (Figure 5B). Our FISH and BLAST mapping data indicate that structural genomic differences between homologous chromosomal elements of *An. albimanus* and *An. gambiae* are caused by multiple evolutionary rearrangements. Our comparison of gene orders with outgroup species *Aedes aegypti* showed that e2/e4 centromere shifting took place in the *An. albimanus* lineage, but the e3/e5 centromere movement did not occur in the *An. albimanus* lineage because this species preserved the ancestral centromere position.

### A physical map for the An. albimanus genome

Our physical mapping via FISH placed 31 of the total 204 scaffolds to polytene chromosomes of *An. albimanus*. Considering both original and split misassembled scaffolds, we have mapped 40 scaffolds with a total length of 167,376,416 bp (Table S3). The remaining 173 scaffolds and unmapped sequences from the gaps between misassembled fragments together make up only 3,131,899 bp (Table S4). The chromosomal position and orientation of the mapped scaffolds are shown in Figure 2. As in *An. gambiae*, we considered the positive orientation of the genome from the telomere end to the centromere end for chromosomes X, 2R, and 3R. The positive orientation of the genome for arms 2L and 3L goes from centromere to telomere. Due to the large N50 scaffold size (18.1 Mb) of the *An. albimanus* assembly, it was possible to assign 98.2% of the genome to polytene chromosomes. The total sizes of mapped genomic scaffolds in each chromosomal arm proportionally correspond to their lengths (Table 2). The largest scaffold, KB672286, has a length of 30.8 Mb and covers the majority of chromosomal arm 3L from region 38A to 45B. Three much smaller scaffolds cover the rest of 3L. Arm 2L is completely mapped by only three large scaffolds. Twelve smaller scaffolds comprise sequences of 3R. Similarly, assemblies of 2R and X consist of multiple short scaffolds. Euchromatic regions of all chromosomes are completely covered on our map with the exception of small gaps in region 4C on X and region 13A on 2R. The unmapped 173 scaffolds are expected to reside in those gaps and in the pericentromeric heterochromatin of all chromosomes. Information about scaffold adjacencies is given in Table S5.

**Table 2:** Proportions of *An. albimanus* polytene chromosomes and mapped genome

	Chromosome X	Arm 2R	Arm 2L	Arm 3R	Arm 3L	Total
Average length, µm	58.6	244.8	167.4	161	161	792.8
Relative length, %	7.4	30.9	21.1	20.3	20.3	100.0
Mapped genome, Mb	11.8	51.3	38.0	32.7	33.6	167.4
Proportion of mapped genome, %	7.1	30.6	22.7	19.5	20.1	100

**Figure 6:**
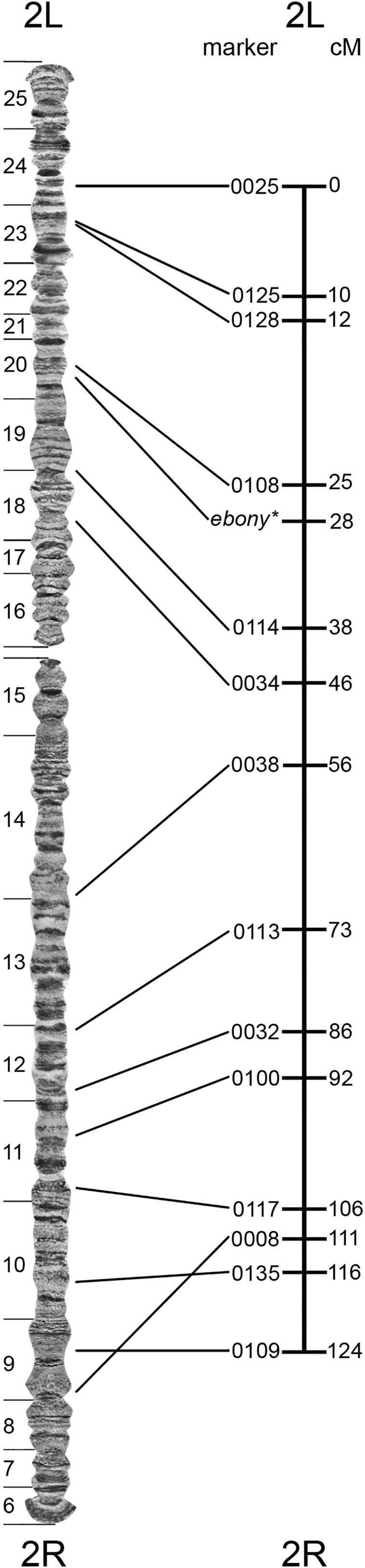
A comparison between the physical (left) and genetic (right) maps of the *An. albimanus* chromosome 2. Lines connect positions of the same markers on the physical and genetic maps.

To assess the degree of correspondence between physical and genetic mapping, we compared the order of scaffolds in our physical map with the order of genetic markers in the linkage map for chromosome 2 published earlier (Penilla *et al.* 2009). The analysis revealed a good correspondence in the position of markers between the two maps. However, the position of microsatellite 0008 in the linkage map contradicted the location of the homologous sequence on the physical map (Figure 6). Some discrepancies between physical and genetic maps can be seen in other comparisons, and they are often associated with local variation in genetic recombination (Timoshevskiy *et al.* 2013; Juneja *et al.* 2014; Shearer *et al.* 2014). For *An. albimanu*s, we observe greater distances between the markers around the centromere on its physical map and smaller distances between them on its genetic map. This difference is likely caused by the reduction of crossing over in pericentromeric regions.

## Discussion

### A high-resolution cytogenetic map is a critical tool for physical genome mapping

Before proceeding with physical mapping, we constructed a new cytogenetic map for salivary gland polytene chromosomes of *An. albimanus* (Figure 2). This step was necessary because the previously published drawn map (Keppler *et al.* 1973) and photomap (Cornel and Collins 2000) were not designed for genome-wide physical mapping. The cytogenetic photomap constructed in this study has three distinct features that make it superior for cytogenetic genome mapping. First, chromosomes are completely straightened and flattened so that banding patterns along the chromosomal arms are not obscured. Second, major cytogenetic landmarks are described in detail making identification of chromosomal arms easier. Third, the photomap consists of high-resolution images of well-polytenized chromosomes, which increases the precision of probe localization. The improved cytogenetic map allowed identification of adjacencies of genomic scaffolds (Table S5).

### Anopheles albimanus has the most complete chromosome-anchored genome map developed for any mosquito

The genome portion mapped in this study represents 98.2% of the total *An. albimanus* genome assembly. For comparison, the physically mapped portion of the *An. gambiae* assembly is 84.3% (https://www.vectorbase.org/organisms/anopheles-gambiae/pest/agamp4) (Sharakhova *et al.* 2007). In our previous study, physical mapping assigned 62% of the genome onto chromosomes of the Indian strain of *An. stephensi* (Jiang *et al.* 2014). The main reason for the success in the near completion of physical genome mapping for *An. albimanus* is the greater length of scaffolds in this species’ assembly. The *An. albimanus* genome assembly consisted of only 204 scaffolds with an N50 scaffold size of 18.1 Mb (Neafsey *et al.* 2015). A genome with longer scaffolds requires fewer FISH experiments for mapping. The most important factor driving the increase of the scaffold length in *An. albimanus* was the reduced genetic heterozygosity in this species, which is lower than in any other sequenced *Anopheles* species (Daniel Neafsey, personal communication). The source for genomic DNA was an isofemale subcolony from MR4, which probably played a role in helping to reduce heterozygosity and thus build a better assembly. Another factor influencing the greater scaffold length in *An. albimanus* was the lower repeat content (only 2%), relative to all sequenced species of *Anopheles* (Neafsey *et al.* 2015; Sharakhov and Sharakhova 2015).

Interestingly, longer scaffolds in *An. albimanus* have not resulted in a 100% accurate genome assembly. The HMM approach identified eight misassemblies in six scaffolds (Table S2). In eight out of nine cases identified by physical mapping, misassemblies occurred in gaps between genomic contigs suggesting that the scaffolding algorithm made mistakes in bridging contigs. Our repeat masking analysis has not detected repetitive sequences at boundaries of misassembled fragments. One misassembly that happened within contig APCK01000384.1 of scaffold KB672397 is likely caused by the sequence similarity among multiple tRNA genes present within the area of misassembly. The physical mapping method found nine misassemblies within five scaffolds (Table 1 and Table S1). The discrepancies between the two approaches come from two sources, both of which are explained by the fact that the HMM method relied on chromosomal location of orthologs in *An. gambiae* and orthology information in other *Anopheles* species. First, the bioinformatics approach missed three misassemblies in two scaffolds, KB672287 and KB672397, which were split by physical mapping within the same chromosomal element. Second, the HMM approach erroneously predicted that two scaffolds, KB672353 and KB672375, might contain misassemblies, which, after considering the evidence, were deemed to be true translocations rather than actual assembly errors (Figure 4). In the majority of cases, when the physical mapping and the HMM method agreed, they together identified six misassemblies in four scaffolds. Physical mapping alone identified three additional misassemblies (Table 1). These results demonstrate how a bioinformatics approach can work synergistically with physical mapping to systematically identify and correct misassemblies in genomic scaffolds. The misassemblies identified computationally certainly could have been identified using FISH alone, but the process of identifying boundaries between fragments within scaffolds would have been much more time consuming. As chromosomal assemblies are developed for more *Anopheles* species, the predictive power of ortholog positions within related species will become a useful tool for the initial identification of genome misassemblies.

A recent study applied the breakpoint graphs algorithm to decrease the *An. albimanus* genome fragmentation (Neafsey *et al.* 2015). This algorithm is based on gene order and genome rearrangement analysis (Aganezov *et al.* 2015). Our *in situ* hybridization experiments tested the following predicted adjacencies: KB672457–KB672404, KB672287B–KB672364–KB672405, KB672409–KB672353, and KB672411–KB672408 (Table S5). We confirmed four immediate scaffold adjacencies that have been predicted by the breakpoint graphs algorithm in *An. albimanus*. Although KB672364 and KB672405 are neighboring scaffolds in the physical map, a gap between them can be seen in our cytogenetic map (Figure 2). This gap resides in the region of intercalary heterochromatin (3R:34B) suggesting that repetitive DNA sequences or *An. albimanus* specific genes could be mapped there.

### Partial-arm translocations cause interarm genetic exchange in genus Anopheles

All previous comparative studies of cytogenetic and physical maps in malaria mosquitoes have come to the conclusion that paracentric inversions and whole-arm translocations are the only large-scale rearrangements that happen in the evolution of *Anopheles* genomes (Green and Hunt 1980; Cornel and Collins 2000; Sharakhov *et al.* 2001, 2002; Xia *et al.* 2010; Sharakhova *et al.* 2011, 2013, 2014; Jiang *et al.* 2014; Liang *et al.* 2014; Artemov *et al.* 2015; Neafsey *et al.* 2015). This conclusion implies that although genes within a chromosomal element “travel” together in evolution they can occasionally move to other elements via transpositions. Our present study demonstrated that the extent of gene exchange between chromosomal arms in the evolution of *Anopheles* can be much more dramatic. This finding has become possible because of the availability of extensive genome mapping data in pericentromeric regions of *An. albimanus* and *An. gambiae*. We have noticed the contradiction between mapping outputs of scaffolds KB672353 and KB672375 from the HMM method and FISH (Figure 4). The HMM approach showed that scaffolds KB672353 and KB672375 each consist of two fragments that belong to different chromosomal elements in *An. gambiae*. Instead, FISH placed each scaffold entirely within one chromosomal element in *An. albimanus*. The BLAST results indicated that gene content differs between pericentromeric regions of homologous chromosomal arms of *An. albimanus* and *An. gambiae*. Our data suggest that the position of centromeres changed by interarm rearrangements during mosquito evolution (Figure 5). The lack of large-scale inverted sets of genes located near breakpoints in these species means that the centromere movements have not been caused by pericentromeric inversions. Instead, numerous partial-arm translocations could disrupt arm integrity near centromeres and locally reshuffle pericentromeric sequences from different chromosomal elements. Therefore, the notion of whole-arm translocations occurring in the evolution of *Anopheles* (Neafsey *et al.* 2015) has to be refined with the notion of partial-arm or near whole-arm translocations. The breakpoints of these translocations can be located at different distances from centromeres (from several hundreds of kilobases to >1 Mb) in different mosquito lineages. As a result, the genetic content near centromeres was reshuffled between chromosomal elements during the 100 million years of evolution in malaria mosquitoes.

### Conclusions

The genome map developed in this study for the Neotropical malaria vector *An. albimanus* demonstrates the power of integrating cytogenetics and physical mapping with synteny information from related species. Using this combined approach, we were able to generate a high-coverage physical genome map for *An. albimanus* that surpasses the mapping coverage of the best-studied malaria mosquito *An. gambiae*. Our study identified and corrected nine scaffold misassemblies, thus highlighting the importance of physical mapping for creating an accurate genome assembly. This combined approach can be applied to improving genome assemblies in other malaria mosquito species. The identification of contradictions between bioinformatics and FISH-based methods revealed rearrangements of gene order in the pericentromeric regions caused by partial-arm translocations. The physical genome map for *An. albimanus* will serve as a convenient outgroup for phylogenetic reconstruction based on fixed inversions in subgenera Anopheles and Cellia and for studying chromosomal evolution in the genus *Anopheles*. Also it can be used as a reference genome map for population genetics studies of the Neotropical malaria vector.

## Supplementary Material

Supplemental material is available online at www.g3journal.org/lookup/suppl/doi:10.1534/g3.116.034959/-/DC1.
