# Supplementary material for: The Physical Genome Mapping of Anopheles albimanus Corrected Scaffold Misassemblies and Identified Interarm Rearrangements in Genus Anopheles
Source: G3 (Bethesda). 2016 Nov 7;7(1):155–64. doi: 10.1534/g3.116.034959 (PMC5217105; doi:10.1534/g3.116.034959)
Supplement: Supplementary file 1 [file 155TableS1.docx]

Table S1. Misassembies in genomic scaffolds of *An. albimanus* identified by the physical mapping, HMM model, and synteny analysis. (.xlsx, 12 KB)

<http://www.g3journal.org/lookup/suppl/doi:10.1534/g3.116.034959/-/DC1/TableS1.xlsx>
