# Supplementary figures and images for: The Physical Genome Mapping of Anopheles albimanus Corrected Scaffold Misassemblies and Identified Interarm Rearrangements in Genus Anopheles
Source: G3 (Bethesda). 2016 Nov 7;7(1):155–64. doi: 10.1534/g3.116.034959 (PMC5217105; doi:10.1534/g3.116.034959)

**Table S2** Misassembies in genomic scaffolds of An. albimanus predicted by the HMM.


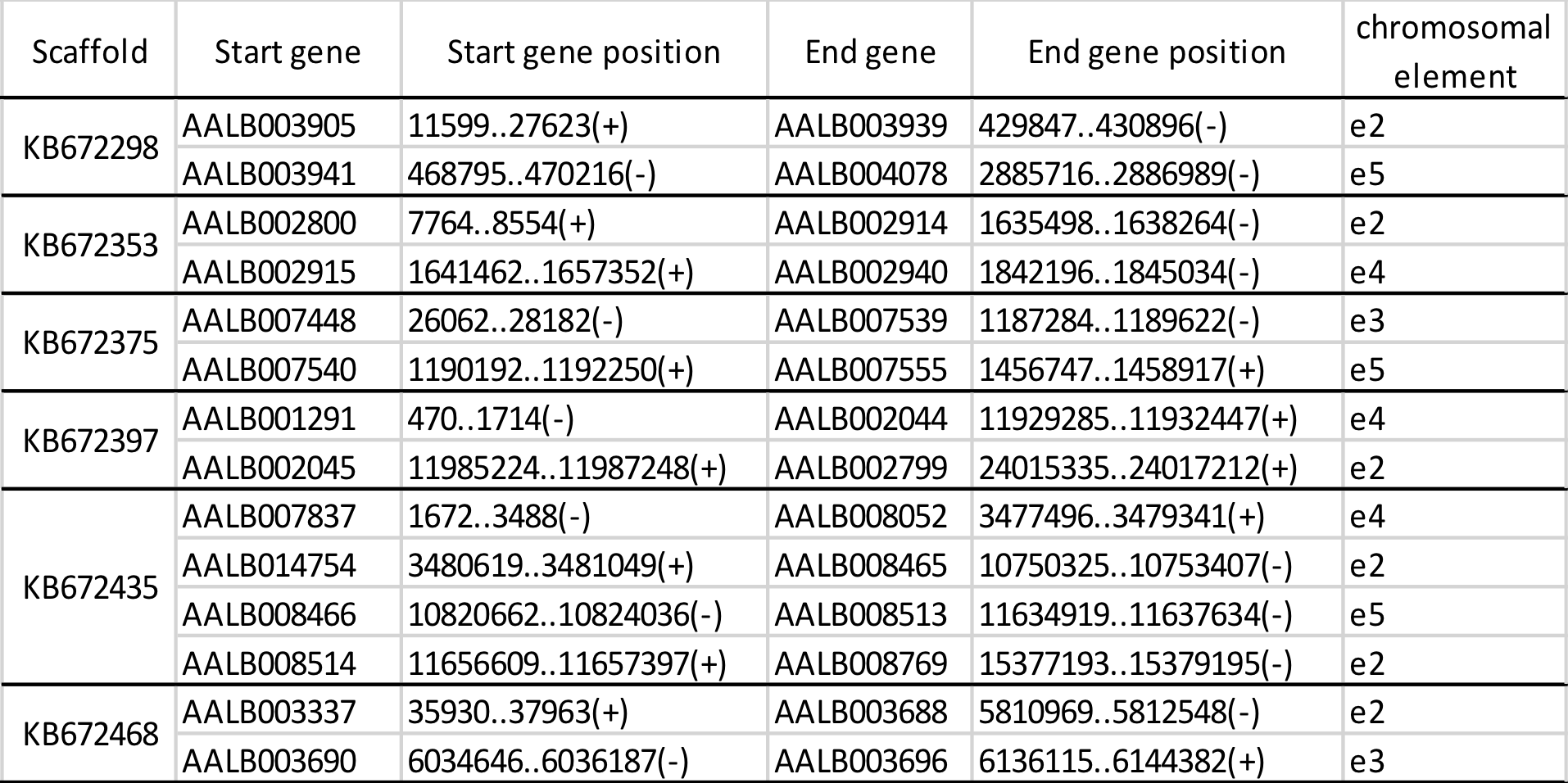

Supplement: Supplementary file 3 [file 155TableS2.docx]
